# Long-term Evaluation of Allogeneic Bone Marrow-derived Mesenchymal Stromal Cell Therapy for Crohn’s Disease Perianal Fistulas

**DOI:** 10.1093/ecco-jcc/jjz116

**Published:** 2019-06-14

**Authors:** Marieke C Barnhoorn, Martin N J M Wasser, Helene Roelofs, P W Jeroen Maljaars, Ilse Molendijk, Bert A Bonsing, Liesbeth E M Oosten, Gerard Dijkstra, C Janneke van der Woude, Dave L Roelen, Jaap-Jan Zwaginga, Hein W Verspaget, Willem E Fibbe, Daniel W Hommes, Koen C M J Peeters, Andrea E van der Meulen-de Jong

**Affiliations:** 1 Department of Gastroenterology and Hepatology, Leiden University Medical Center, Leiden, The Netherlands; 2 Department of Radiology, Leiden University Medical Center, Leiden, The Netherlands; 3 Department of Immunohematology and Blood Transfusion, University Medical Center, Leiden, The Netherlands; 4 Department of Surgery, Leiden University Medical Center, Leiden, The Netherlands; 5 Department of Gastroenterology and Hepatology, University Medical Center Groningen, Groningen, The Netherlands; 6 Deparment of Gastroenterology and Hepatology, Erasmus MC, Rotterdam, The Netherlands

**Keywords:** Mesenchymal stromal cells, Crohn’s disease, perianal fistulas

## Abstract

**Background and Aims:**

The long-term safety and efficacy of allogeneic bone marrow-derived mesenchymal stromal cell [bmMSC] therapy in perianal Crohn’s disease [CD] fistulas is unknown. We aimed to provide a 4-year clinical evaluation of allogeneic bmMSC treatment of perianal CD fistulas.

**Methods:**

A double-blind dose-finding study for local bmMSC therapy in 21 patients with refractory perianal fistulising Crohn’s disease was performed at the Leiden University Medical Center in 2012–2014. All patients treated with bmMSCs [1 x 10^7^ bmMSCs cohort 1, *n* = 5; 3 × 10^7^ bmMSCs cohort 2, *n* = 5; 9 × 10^7^ bmMSCs cohort 3, *n* = 5] were invited for a 4-year evaluation. Clinical events were registered, fistula closure was evaluated, and anti-human leukocyte antigen [HLA] antibodies were assessed. Patients were also asked to undergo a pelvic magnetic resonance imaging [MRI] and rectoscopy.

**Results:**

Thirteen out of 15 patients [87%] treated with bmMSCs were available for long-term follow-up. Two non-MSC related malignancies were observed. No serious adverse events thought to be related to bmMSC therapy were found. In cohort 2 [*n* = 4], all fistulas were closed 4 years after bmMSC therapy. In cohort 1 [*n* = 4] 63%, and in cohort 3 [*n* = 5] 43%, of the fistulas were closed, respectively. In none of the patients anti-HLA antibodies could be detected 24 weeks and 4 years after therapy. Pelvic MRI showed significantly smaller fistula tracts after 4 years.

**Conclusions:**

Allogeneic bmMSC therapy for CD-associated perianal fistulas is also in the long-term a safe therapy. In bmMSC-treated patients, fistulas with closure at Week 24 were still closed after 4 years.

## 1. Introduction

A serious, often persistent, complication of Crohn’s disease [CD], occurring in 25% of patients, is the development of perianal fistulas.^1,2^ Perianal fistulas are ulcer tracts that connect the intestinal lumen, from the anal canal or rectum, with the perianal skin and are associated with a strongly impaired quality of life. Although healing of luminal ulcers can be achieved, complete fistula healing in CD is difficult and is accompanied by multiple relapses. The combination of both biologic therapies and fistula drainage with a non-cutting seton is still the cornerstone of treatment. After adequate drainage, closure of the tract can be performed using the advancement flap or the ligation procedure of the intersphincteric tract. Faecal diversion is considered one of the last treatment options, with a first response rate of 64%.^3^ Of the biologic therapies, only infliximab and adalimumab have been found effective in randomised controlled trials for the closure of perianal fistulas in CD till now.^4–6^ In the end, only 37% of the patients with complex perianal fistulas showed fistula closure after a median follow-up of 10 years using combined medico-surgical therapies.^7^ These disappointing healing rates show the need for new therapies for perianal fistulising CD.

A promising therapy for perianal CD fistulas is the local injection of bone marrow-derived mesenchymal stromal cells [bmMSCs].^8^ In 2012–2014, we conducted a randomised placebo-controlled dose-finding study for the treatment of perianal CD fistulas with allogeneic bmMSCs.^9^ That study, with a follow-up of 24 weeks, showed that locally administered bmMSCs for perianal CD fistulas were safe and feasible. Furthermore, the study showed a significant improvement of fistula closure in patients treated with 3 x 10^7^ bmMSCs [cohort 2] compared with placebo-treated patients, with a reduction in the number of draining fistulas of 86%. These promising results have been confirmed in a large multicentre trial from Panes *et al*.^10^ In that study, fistula closure was reached at Week 24 in 50% [*n* = 53] of the 107 patients receiving local adipose tissue-derived MSCs [Cx601; 12 × 10^7^ MSCs] versus 34% [*n* = 36] in the 105 placebo-treated patients. In 2017, Cx601 was approved by the European Medicines Agency for the treatment of complex perianal fistulizing CD. Here we report on long-term safety and efficacy of local bmMSC therapy in CD perianal fistulas.

## 2. Materials and Methods

### 2.1. Study design

We asked all patients enrolled in the randomised placebo-controlled dose-finding trial ‘Allogeneic bone marrow-derived mesenchymal stem cells for the treatment of fistulas in patients with refractory perianal Crohn’s disease’ [NCT01144962; clinicaltrials.gov] for a 4-year follow-up evaluation. Full details of the original study design, the patient eligibility criteria, and the primary outcome of the study after 24 weeks of follow-up have been published previously^9^ [Supplementary Figure 1, available as Supplementary data at *ECCO-JCC* online]. In short, 21 patients with refractory perianal fistulising CD were enrolled. Patients were double-blind randomised in a 5:2 fashion to receive locally either 0.9% NaCl/5% human albumin solution solution with 1 × 10^7^ [cohort 1, *n* = 5], 3 × 10^7^ [cohort 2, *n* = 5], or 9 × 10^7^ [cohort 3, *n* = 5] bmMSCs or solution with no cells [placebo group, *n* = 6]. Before local bmMSC or placebo injection, the surgeon performed curettage of the fistula tract[s], the mucosa or skin of, respectively, the internal and external opening, and the internal opening with an absorbable polydioxanone II 4/0 suture. Subsequently, half of the bmMSCs or placebo suspension was injected via the anus in the fistula wall around the closed internal opening. The second half was injected in the wall as close as possible to the internal opening by introducing the syringe into the fistula tract via the external opening.

Four years after treatment in the clinical trial, patients who received bmMSC therapy were asked to visit the outpatient clinic, and placebo-treated patients were consulted by phone. Patients treated with bmMSCs were asked for clinical events and the clinical fistula closure was evaluated [e.g., no fistula discharge]. Furthermore, patients were asked to fill out questionnaires concerning current medication use, operation history, the Perianal Disease Activity Index [PDAI], adapted Vaizey faecal incontinence score, Crohn’s Disease Activity Index [CDAI], Short Form [SF]-36 score, and Short Inflammatory Bowel Disease Questionnaire [sIBD-Q]. The CDAI and Vaizey score were not calculated in two patients with a stoma. All bmMSC-treated patients were also asked to undergo a rectoscopy and pelvic magnetic resonance imaging [MRI] 4 years after MSC therapy. Pelvic MRI scans before bmMSC therapy and 4 years after therapy were evaluated by an experienced radiologist [MNJMW]. The diameter of the fistula tract[s] and the presence of collections were reported. Improvement on MRI was defined by fistulas containing less fluid compared with the MRI scan made before bmMSC therapy. Furthermore, if possible, blood was drawn for standard measurements and serum collection.

The original study and the amendment for this follow-up study were approved by the Central Committee on Research involving Human Subjects and the local Medical Ethical Committee of Leiden University Medical Center. All patients gave a renewed written informed consent for the follow-up study. Data were collected between May 2016 and May 2018. All authors had access to the study data and reviewed and approved the final manuscript.

### 2.2. Human leukocyte antigen antibody measurements

In the serum of patients treated with bmMSCs, donor-specific antibodies against human leukocyte antigen [HLA] class I and II were measured using the Luminex Screening Assay: Lifecodes Lifescreen Deluxe [LMX] kit according to the manufacturer’s manual [Immucor Transplant Diagnostics Inc., Stamford, CT, USA]; the modified protocol as described by Kamburova *et al*.^11^ was used. Provider-suggested definitions of the negative and positive discriminations were used. When positive, donor specificity was determined by single antigen bead assay [Luminex single-antigen [LSA], Lifecodes] according to the manufacturer’s protocol. Sera were pre-treated with EDTA before testing in LSA. All Luminex tests were analysed with a Luminex 100 reader. First the serum collected 24 weeks [from two patients only Week 12 serum was available] after bmMSC therapy was measured. When no antibodies were found after 24 weeks, serum samples 4 years after bmMSC therapy were tested when available.

### 2.3. Statistical analysis

Paired data [before and 4 years after bmMSC therapy] were compared using the Paired t-test. Data were analysed using SPSS statistical software package [version 23, IBM SPSS Statistics for Windows, IBM Corp, Armonk, NY] or GraphPad Prism software [version 7, San Diego, CA] and expressed as means ± SEM. *p* ≤ 0.05 was considered statically significant.

## 3. Results

### 3.1. Study population

Two of the 15 bmMSCs-treated patients were not available for long-term follow-up [Table 1]. One patient in cohort 1 died due to an adenocarcinoma in the caecum, which was already described in the original paper of the study,^9^ and in cohort 2 one patient was lost to follow-up. Six patients received placebo, of whom two patients received open-label bmMSC therapy in our centre 2 years after the initial study, and one patient was treated with Cx601^10^ 2 years later. These three patients had draining fistula[s] at the time of these treatments. The other three placebo-treated patients were consulted by phone for evaluation of fistula drainage. Medication use at the time of the follow-up visit and at surgery in the past 4 years are described in Table 2.

**Table 1.** Study population.

Cohort	bmMSCs	Treated patients [*n*]	Patients in follow-up [*n*]	Original treated fistulas [*n*]^d^	MRI [*n*]	Rectoscopy [*n*]
1	1 × 10^7^	5	4	8	3	3
2	3 × 10^7^	5	4^a^	6	3	2
3	9 × 10^7^	5	5^b^	7	3	1
Placebo	-	6	3^c^	3	-	-

MRI, magnetic resonance imaging.

^a^One patient was consulted by phone due to emigration.

^b^One patient was consulted by phone due to long travel time.

^c^All with placebo-treated patients were consulted by phone.

^d^In patients included in the follow-up.

**Table 2.** Medication use and surgery. Medication use at the time of the 4-year follow-up visit and surgery during past 4 years in bmMSC-treated patients.

	Cohort 1 [*n* = 4]	Cohort 2 [*n* = 4]	Cohort 3 [*n* = 5]
Age at follow-up, mean, years [range]	43 [31–57]	46 [43–51]	38 [26–52]
Male, *n* [%]	3 [75%]	4 [100%]	1 [20%]
Medication, *n* [%]			
No medication	1 [25%]	1 [25%]	2 [40%]
IFX / ADA	2 [50%]	2 [50%]	2 [40%]
VED	1 [25%]	1 [25%]	-
Thiopurines/MTX only	-	-	1 [20%]
Surgery, *n* [%] in past 4 years			
I and D	2 [1]	-	3 [3]
Ileocaecal resection	-	1 [1]	-
Rectum extirpation	-	-	1 [1]
Setons in situ	1 [25%]	-	1 [20%]

IFX, infliximab; ADA, adalimumab; VED, vedolizumab; MTX, methotrexate; I and D, incision and drainage.

### 3.2. Safety

Clinical events over 4 years after bmMSC therapy were assessed. Four patients had developed perianal abscesses, three patients had CD activity in the past 4 years, and five patients were treated for infections [Table 3]. During the 4-year follow-up visit, we detected B-cell lymphoproliferative disease [LPD] in the rectum of a patient treated with 3 × 10^7^ bmMSCs [cohort 2] as reported previously.^12^ To investigate whether bmMSC therapy contributed to this LPD, we analysed the possibility of Epstein-Barr virus [EBV] transfer via the MSC product and the persistence of bmMSCs in the LPD tissue, using short tandem repeat analysis. Since no EBV-DNA was detected in the bmMSC product and no cells containing the DNA of the MSC donor were detected in the lymphoproliferative lesion, it was concluded that a relation between this EBV-associated LPD and the bmMSC therapy was unlikely, but rather was the result of prolonged immunosuppressive therapy.

**Table 3.** Clinical events in the past 4 years in bmMSC-treated patients included in the long-term follow-up. Number of adverse events in total, [in number of patients].

	Cohort 1 [*n* = 4]	Cohort 2 [*n* = 4]	Cohort 3 [*n* = 5]
Perianal abscess	2 [1]		4 [3]
Activity CD^a^	1 [1]	1 [1]	1 [1]
Infection^b^	4 [2]	1 [1]	2 [2]
Gout	1 [1]		
Psoriasis guttae	1 [1]		
Uveitis		2 [1]	
Malignancy^c^		1 [1]	

CD, Crohn’s disease.

^a^Including diversion proctitis.

^b^Pneumonia, otitis media, fungal infection, periodontal abscess, laryngitis.

^c^B-cell lymphoproliferative disease.

### 3.3. Efficacy

**Figure 1.**
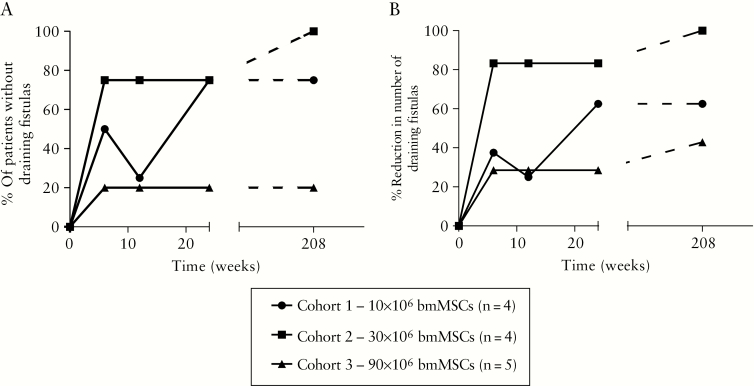
Fistula closure after 4 years of follow-up. [A] Percentage of patients per group without draining fistulas at Weeks 6, 12, 24, and 208 after therapy. [B] Percentage of reduction in the number of draining fistulas per group at Weeks 6, 12, 24, and 208 after therapy. Only patients who were evaluated in the long-term follow-up were included in the graphs.

The beneficial effect of bmMSC therapy on the number of draining fistulas previously reported at Week 24 was maintained after 4 years [Figure 1; and Supplementary Table 1, available as Supplementary data at *ECCO-JCC* online]. In cohort 1, 75% [3/4] of the patients had complete clinical fistula closure after 4 years, as determined by the absence of discharge. In cohort 2, all patients (100% [4/4]) had complete clinical fistula closure after 4 years. In contrast, in cohort 3, only one (20% [1/5]) patient had complete clinical fistula closure; however, two patients showed partial fistula closure, with closure of either one of their two fistulas (40% [2/5]). In two patients [cohorts 2 and 3] perianal fistulas closed between Week 24 and the 4-year follow-up without surgical interventions in the perianal region. In the three patients in the placebo group, none of the patients experienced partial or complete fistula closure at 4 years (0% [0/3]). The bmMSCs from one donor [bmMSC-B] showed lower fistula healing rates compared with the bmMSCs of the other four MSC donors [Supplementary Figure 2, available as Supplementary data at *ECCO-JCC* online].

**Figure 2.**
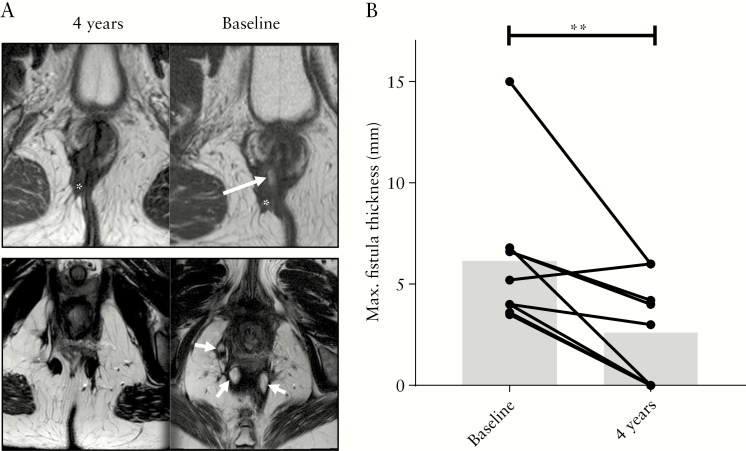
bmMSC therapy causes fistula closure confirmed on pelvic MRI. [A] Magnetic resonance images [MRI] of the perianal region of two patients treated with bmMSC therapy. Above: images at baseline and after 4 years from a patient treated in cohort 2. The fistula tract is completely closed, with only scar tissue being present 4 years after bmMSC therapy. Under: images at baseline and after 4 years from a patient treated in cohort 3. Fistula tracts that contained fluid before bmMSC therapy are closed 4 years later. Arrows: fluid inside the fistula tracts. Asterisks: scar tissue. [B] Maximum fistula diameter [mm] at baseline and 4 years after MSC therapy [*n* = 9]; **p <0.01.

MRI evaluation after 4 years showed an improvement in the fistula tracts in 67% [6/9] of MSC treated patients [Figure 2A]. In only four patients (44% [4/9]) complete fibrotic fistula tracts were seen. The maximal fistula diameter found in all treated fistulas before bmMSC therapy and 4 years thereafter was measured and showed significant improvement [6.1 mm ± 1.2 vs 2.6 mm ± 0.9, *p* = 0.006] [Figure 2B]. In none of the scans the presence of >2-cm collections was demonstrated, although in two patients smaller abscesses were observed. No de novo fistulas were found.

**Figure 3.**
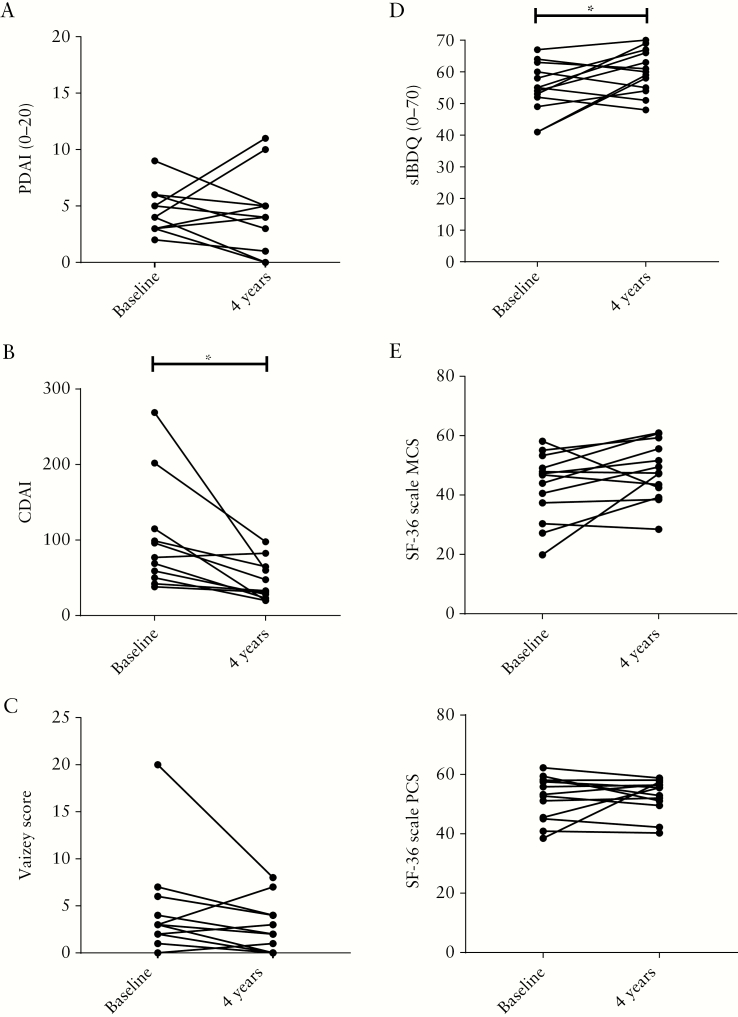
Improvement of quality of life after bmMSC therapy. [A] Perianal Disease Activity Index [PDAI] before and 4 years after bmMSC therapy [*n* = 13]. [B] Crohn’s Disease Activity Index [CDAI] before and 4 years after bmMSC therapy [*n* = 11]. [C] Vaizey score for incontinence before and 4 years after bmMSC therapy [*n* = 11]. [D] Short Inflammatory Bowel Disease Questionnaire [sIBDQ] before and after bmMSC therapy [*n* = 13]. [E] Mental [MCS] and physical [PCS] component score of the Short Form [SF] 36 before and after bmMSC therapy [*n* = 13]; *p <0.05.

For each patient, the PDAI, CDAI, Vaizey score and quality of life measurements were compared between baseline and 4 years later. In nine out of 13 patients, PDAI scores 4 years after bmMSC treatment were found to be lower than before bmMSC therapy, which however did not reach statistical significance [4.3 vs 3.8, *p* = 0.585] [Figure 3A]. The CDAI revealed a significantly lower disease activity 4 years after bmMSC therapy [46.2 vs 101.5 *p* = 0.014] [Figure 3B]. No difference between the Vaizey score at baseline and after 4 years follow-up was found [Figure 3C]. Comparison of the SF-36 and sIBDQ before and 4 years after bmMSC therapy showed that bmMSC-treated patients had improvement of their quality of life [sIBDQ: 54.8 vs 60.1, *p* = 0.047; SF-36 MCS: 42.8 vs 48.1, *p* = 0.089; SF-36 PCS: 52.2 vs 52.8, *p* = not significant] [Figure 3D and E].

### 3.4. Anti-HLA antibodies

In sera of patients included in the trial, we measured class I and II anti-human leukocyte antigen [HLA] antibodies, but none could be detected after 24 weeks [*n* = 15] and 4 years of therapy [*n* = 9].

## 4. Discussion

Perianal fistulas remain a common and challenging complication of CD, with limited treatment options and invalidating complaints. In this follow-up study, we evaluated the long-term safety and efficacy of bmMSC therapy as a new therapy for CD-associated perianal fistulas; bmMSC therapy was already shown to be a safe and efficacious option for short-term closure of perianal fistulas in both our^9^ and other clinical trials.^10, 13–16^ The current study revealed that bmMSC therapy is also safe and efficacious in the long-term.

The results after 24 weeks^9^ showed that in cohorts 1, 2, and 3 respectively, 67%, 86%, and 29% of the perianal fistulas were closed versus 33% in the placebo group. In this long-term follow-up, we found fistula closure rates of 63%, 100%, and 43% after 4 years in the three different MSC cohorts. Therefore we concluded that fistula healing persisted. In contrast, in the the placebo-treated patients, all fistulas were draining [again] after 4 years. Next to clinical evaluation of fistula closure, we found improvement of the fistula tracts in 67% of the bmMSC-treated patients on pelvic MRI. However, complete fibrosis of fistula tracts was only observed in 44% of the patients. These results are in line with previous reports, showing that fistula tracks persisted on MRI despite [long-term] clinical remission.^17^ Most of the patients evaluated in this long-term follow-up study experienced fistula closure already before 24 weeks of bmMSC treatment, but in two patients the fistula[s] closed only after 24 weeks [cohorts 2 and 3]. However, due to the low number of patients in our study and the loss of two patients in the long-term follow-up, caution is advised interpreting these results.

Besides high fistula closure rates in bmMSC-treated patients, we also observed a significantly higher quality of life in bmMSC-treated patients 4 years after therapy compared with their own baseline results. This endpoint is important, since this directly reviews the effect of fistula closure on daily life. However, we also found a lower CDAI in bmMSC=treated patients, suggesting that these patients had an overall lower CD activity 4 years later, which could also result in a better quality of life. In the future, it would be interesting to take work productivity and life style restriction into account when evaluating bmMSC therapy for perianal fistulas.

Although so far no major safety concerns have been raised in previous clinical trials using MSC therapy,^18^ long-term safety aspects should always be evaluated carefully when using cell therapy. In this 4-year follow-up, one patient developed Epstein-Barr virus [EBV]-positive B-cell proliferative disease in the rectum 4 years after treatment with EBV-negative MSCs, which is described in a case report.^12^ In the end it was concluded that this LPD was not related to bmMSC therapy, but more likely was the result of prolonged immunosuppressive therapy since this patient used azathioprine, adalimumab, and methotrexate in the past, and currently vedolizumab. However, the possibility of additional local immunosuppression by bmMSC therapy cannot be discarded completely. Furthermore, as already earlier described and judged not related to the bmMSCtherapy,^9^ one patient in our study died due to an adenocarcinoma in the caecum 2 years after treatment. Notwithstanding their complete different origin and without any expected relation to MSC therapy, these encountered malignancies warrant at least prudent [pre]-selection of patients and continuous long-term monitoring of local MSC therapy.

To date, only a few papers evaluated the long-term outcome of patients treated locally with MSCs for refractory CD fistulas in terms of safety and efficacy. Ciccocioppo *et al*.^19^ showed that autologous bmMSCs were a safe therapy in eight patients after 72 months of follow-up. The probability of fistula relapse-free survival was 88% after 1 year, 50% after 2 years, and 37% after 4 years in this group of patients. Since it is still under debate whether autologous MSCs of IBD patients could be impaired like MSCs from systemic lupus erythematosus patients,^20^ these disappointing long-term efficacy data could be related to the autologous origin of these cells. Other studies concerning local MSC therapy for perianal fistulas only evaluated results up to 2 years.^21–23^ In this regard, Panes *et al*.^22^ showed local Cx601, MSCs derived from adipose tissue, after 12 months to achieve a significantly higher proportion of fistula closure compared with controls [56% vs 39%].

Interestingly, in our study none of the patients treated with bmMSC therapy developed anti-HLA antibodies after 24 weeks or 4 years. This is in contrast to the treatment with Cx601,^10^ in which 34% [18 of 53] of the MSC-treated patients found negative at baseline generated anti-HLA class I antibodies after MSC therapy. The difference in the percentage of patients who formed anti-HLA antibodies might be explained by the origin of the MSC product, since Cx601 is a product consisting of MSCs derived from adipose tissue. The clinical relevance of the presence of anti-HLA antibodies is not elucidated yet, although no relation with response rates was observed in the Cx601 trial.

Of course, results of bmMSC therapies from different clinical trials can only be properly compared when standardised and validated protocols are being used.^24^ Our proposed protocol includes MRI and rectoscopy to localise and classify the tract[s], and closure of the internal orifice and curettage of the tract directly before bmMSC administration. Only patients without active luminal CD or strictures in the distal colon should be eligible.

In conclusion, we have shown that the efficacy of local bmMSC therapy for perianal CD fistulas was maintained for up to 4 years after treatment. These long-term data show that bmMSC therapy is not only able to heal refractory perianal fistulas in CD patients, but also to improve patients’ quality of life. Although we have carefully judged the serious adverse events reported in this study and concluded that there was no relation with bmMSC treatment, more long-term safety data are needed, from both clinical trials and daily clinical practice, to fully appreciate all safety aspects concerning local bmMSC therapy.

## Supplementary Material

jjz116_suppl_Supplementary_Table

jjz116_suppl_Supplemantery_Figures

jjz116_suppl_Supplemantery_Figures_Legend
