# Supplementary material for: Long-term Evaluation of Allogeneic Bone Marrow-derived Mesenchymal Stromal Cell Therapy for Crohn’s Disease Perianal Fistulas
Source: J Crohns Colitis. 2019 Jun 14;14(1):64–70. doi: 10.1093/ecco-jcc/jjz116 (PMC6930001; doi:10.1093/ecco-jcc/jjz116)
Supplement: jjz116_suppl_Supplementary_Table [file jjz116_suppl_supplementary_table.docx]

**Supplementary Table 1**

|  | **Cohort 1** | **Cohort 2** | **Cohort 3** |
| --- | --- | --- | --- |
| Patients with closed fistulas at 4yr, n (%) | 3/4 (75%) | 4/4 (100%) | 1/5 (20%) |
| Patients with closed fistulas at 24wk, n (%) | 3/4 (75%) | 3/4 (75%) | 1/5 (20%) |
| Closed fistulas at 4yr, n (%) | 5/8 (62.5%) | 6/6 (100%) | 3/7 (42.9%) |
| Closed fistulas at 24wk, n (%) | 5/8 (62.5%) | 5/6 (83.3%) | 2/7 (28.6%) |

Supplementary Table 1. Number of patients in long-term follow-up with closed fistulas per cohort and number of closed fistulas per cohort at 24 weeks and 4 years.
