# Supplementary figures and images for: Long-term Evaluation of Allogeneic Bone Marrow-derived Mesenchymal Stromal Cell Therapy for Crohn’s Disease Perianal Fistulas
Source: J Crohns Colitis. 2019 Jun 14;14(1):64–70. doi: 10.1093/ecco-jcc/jjz116 (PMC6930001; doi:10.1093/ecco-jcc/jjz116)

# Supplementary Figure 1

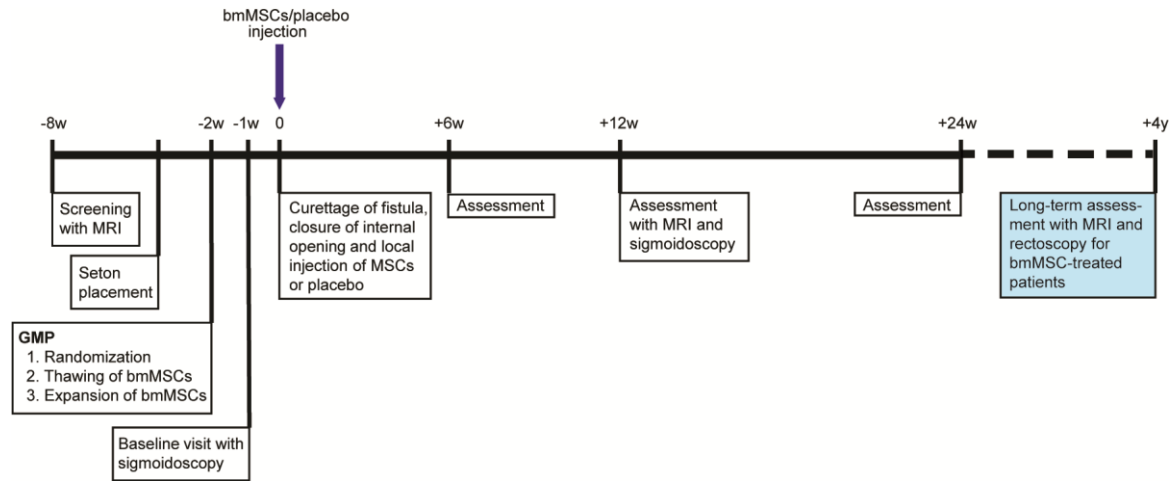

# Supplementary Figure 2

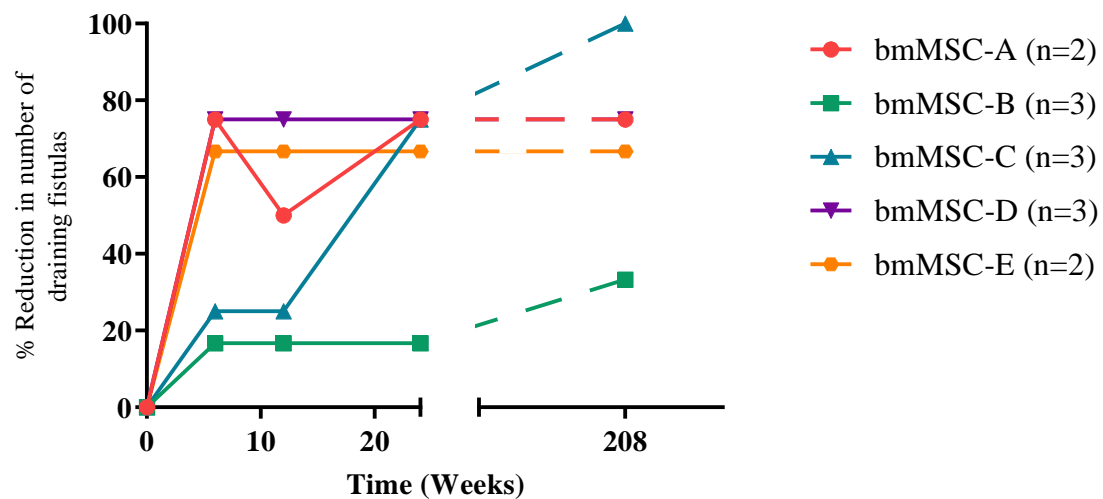

Supplement: jjz116_suppl_Supplemantery_Figures [file jjz116_suppl_supplemantery_figures.pdf]
