# Supplementary material for: Long-term Evaluation of Allogeneic Bone Marrow-derived Mesenchymal Stromal Cell Therapy for Crohn’s Disease Perianal Fistulas
Source: J Crohns Colitis. 2019 Jun 14;14(1):64–70. doi: 10.1093/ecco-jcc/jjz116 (PMC6930001; doi:10.1093/ecco-jcc/jjz116)
Supplement: jjz116_suppl_Supplemantery_Figures_Legend [file jjz116_suppl_supplemantery_figures_legend.docx]

Supplementary Figure 1. Short- and long-term follow up of patients treated in the randomized placebo-controlled dose-finding trial ‘Allogeneic Bone Marrow Derived Mesenchymal Stem Cells for the Treatment of Fistulas in Patients with Refractory Perianal Crohn’s Disease’ [NCT01144962].

Supplementary Figure 2. Fistula closure in relation to bmMSCs from different donors.
